# Supplementary material for: Temporal Trends of Exposure to Organochlorine Pesticides in the United States: A Population Study from 2005 to 2016
Source: Int J Environ Res Public Health. 2022 Mar 24;19(7):3862. doi: 10.3390/ijerph19073862 (PMC8997618; doi:10.3390/ijerph19073862)
Supplement: Supplementary file 1 [file ijerph-19-03862-s001.zip › ijerph-1630439-supplementary.pdf]

Table S1. Maximum Limit of Detection (MLOD) of OCPs by Survey Cycles.

Compound	Survey Cycles					
	2005–2006	2007–2008	2009–2010	2011–2012	2013–2014	2015–2016
β–HCH	1.02[a]	1.00	2.19	0.92	0.92	0.92
HCB	2.72	1.00	3.11	1.7	0.92	0.92
p,p′–DDE	0.45	1.00	3.68	3.1	1.3	0.92
p,p′–DDT	0.88	1.00	2.19	0.92	0.92	0.92
trans–nonachlor	0.45	1.00	2.19	0.92	0.92	0.92
Mirex	1.03	1.00	2.19	0.92	0.92	0.92
Oxychlordane	0.85	1.00	14.14	0.92	0.92	0.92
γ–HCH	1.03	1.00	2.19	0.92	NA	NA
o,p'–DDT	NA	NA	NA	NA	NA	NA

[a]The values given in the tables are the maximum limit of detection (MLOD) divided by the square root of 2 and are expressed as ng/g lipid.

NA: not available or not reported

Table S2. Estimated half–life in humans and log Kow of target OCPs.

Characteristics	β–HCH	HCB	p,p′–DDE	p,p′–DDT	trans–nonachlor
Half-life	7 years	4 – 8 years	8.6 years	2 years	21 – 88 days
Log Kow	3.78	5.73	6.51	6.91	6.20

Table S3. LSGM (ng/g Lipid) and 95% CI by Demographic Groups for HCB

Category	LSGM	95% CI	Pairwise comparison: *P*		
**Sex and Survey (*P* = 0.280)**					
Cycle 2005–2006					
Male	9.62	(9.42, 9.83)			
Female	9.61	(9.44, 9.79)	0.916		
Cycle 2007–2008					
Male	9.49	(9.34, 9.65)			
Female	9.44	(9.31, 9.57)	0.546		
Cycle 2009–2010					
Male	9.37	(9.25, 9.49)			
Female	9.27	(9.16, 9.38)	0.174		
Cycle 2011–2012					
Male	9.24	(9.14, 9.35)			
Female	9.1	(9, 9.21)	0.047		
Cycle 2013–2014					
Male	9.12	(9, 9.24)			
Female	8.94	(8.82, 9.06)	0.039		
Cycle 2015–2016					
Male	8.99	(8.84, 9.15)			
Female	8.78	(8.63, 8.93)	0.054		
**Age Group and Survey (*P* = 0.169)**			12–19 years	20–39 years	40–59 years
Cycle 2005–2006					
12–19 years	7.77	(7.42, 8.13)			
20–39 years	8.11	(7.93, 8.29)	0.378		

40–59 years	10.37	(10.12, 10.62)	< 0.001	< 0.001	
60+ years	13.34	(12.97, 13.73)	< 0.001	< 0.001	< 0.001
Cycle 2007–2008					
12–19 years	7.57	(7.32, 7.83)			
20–39 years	8.02	(7.89, 8.16)	0.018		
40–59 years	10.21	(10.02, 10.4)	< 0.001	< 0.001	
60+ years	13.09	(12.82, 13.38)	< 0.001	< 0.001	< 0.001
Cycle 2009–2010					
12–19 years	7.38	(7.21, 7.55)			
20–39 years	7.94	(7.84, 8.04)	< 0.001		
40–59 years	10.05	(9.91, 10.2)	< 0.001	< 0.001	
60+ years	12.85	(12.65, 13.06)	< 0.001	< 0.001	< 0.001
Cycle 2011–2012					
12–19 years	7.19	(7.07, 7.32)			
20–39 years	7.86	(7.77, 7.95)	< 0.001		
40–59 years	9.9	(9.76, 10.03)	< 0.001	< 0.001	
60+ years	12.61	(12.43, 12.8)	< 0.001	< 0.001	< 0.001
Cycle 2013–2014					
12–19 years	7.01	(6.86, 7.16)			
20–39 years	7.78	(7.67, 7.88)	< 0.001		
40–59 years	9.74	(9.58, 9.91)	< 0.001	< 0.001	
60+ years	12.38	(12.16, 12.6)	< 0.001	< 0.001	< 0.001
Cycle 2015–2016					
12–19 years	6.83	(6.62, 7.05)			
20–39 years	7.7	(7.56, 7.84)	< 0.05		
40–59 years	9.59	(9.39, 9.8)	< 0.001	< 0.001	
60+ years	12.15	(11.87, 12.44)	< 0.001	< 0.001	< 0.001
**Race* and Survey (*P* = 0.007)**			MA	NHB	NHW
Cycle 2005–2006					
MA	11.86	(11.4, 12.33)			
NHB	9.26	(9.06, 9.47)	< 0.001		
NHW	8.3	(8.03, 8.59)	< 0.001	< 0.001	
OTHER	9.66	(9.19, 10.15)	< 0.001	0.45	< 0.001
Cycle 2007–2008					
MA	11.39	(11.06, 11.73)			
NHB	9.13	(8.98, 9.28)	< 0.001		
NHW	8.26	(8.05, 8.47)	< 0.001	< 0.001	
OTHER	9.52	(9.17, 9.89)	< 0.001	0.193	< 0.001
Cycle 2009–2010					
MA	10.95	(10.71, 11.19)			
NHB	8.99	(8.88, 9.11)	< 0.001		
NHW	8.21	(8.07, 8.36)	< 0.001	< 0.001	
OTHER	9.39	(9.14, 9.65)	< 0.001	0.029	< 0.001
Cycle 2011–2012					
MA	10.52	(10.31, 10.73)			
NHB	8.86	(8.77, 8.95)	< 0.001		
NHW	8.17	(8.05, 8.28)	< 0.001	< 0.001	
OTHER	9.26	(9.07, 9.46)	< 0.001	0.002	< 0.001
Cycle 2013–2014					
MA	10.11	(9.86, 10.36)			
NHB	8.73	(8.64, 8.83)	< 0.001		

NHW	8.12	(7.98, 8.26)	< 0.001	< 0.001	
OTHER	9.13	(8.92, 9.34)	< 0.001	0.007	< 0.001
Cycle 2015–2016					
MA	9.72	(9.4, 10.05)			
NHB	8.6	(8.48, 8.73)	< 0.001		
NHW	8.08	(7.88, 8.28)	< 0.001	< 0.001	
OTHER	9	(8.73, 9.29)	< 0.01	0.071	< 0.001

*MA: Mexican American; NHB: Non–Hispanic black; NHW: Non–Hispanic white; OTHER: other than MA, NHB, and NHW

Table S4. LSGM (ng/g Lipid) and 95% CI by Demographic Groups for p, p'–DDE

Category	LSGM	95% CI	Pairwise comparison: *P*		
**Sex and Survey (*P* = 0.182)**					
Cycle 2005–2006					
Male	447.65	(426.75, 469.56)			
Female	441.78	(424.61, 459.65)	0.648		
Cycle 2007–2008					
Male	378.82	(365.21, 392.94)			
Female	378.36	(366.74, 390.35)	0.955		
Cycle 2009–2010					
Male	320.58	(311.72, 329.7)			
Female	324.05	(315.95, 332.34)	0.493		
Cycle 2011–2012					
Male	271.3	(264.59, 278.17)			
Female	277.53	(270.98, 284.24)	0.096		
Cycle 2013–2014					
Male	229.59	(222.96, 236.41)			
Female	237.69	(231.19, 244.36)	0.039		
Cycle 2015–2016					
Male	194.29	(186.95, 201.92)			
Female	203.56	(196.55, 210.83)	0.043		
**Age Group and Survey (*P* = 0.034)**			12–19 years	20–39 years	40–59 years
Cycle 2005–2006					
12–19 years	177.21	(168.1, 186.81)			
20–39 years	297.55	(276.75, 319.92)	< 0.001		
40–59 years	525.69	(503.79, 548.54)	< 0.001	< 0.001	
60+ years	1439.54	(1371.65, 1510.8)	< 0.001	< 0.001	< 0.001
Cycle 2007–2008					
12–19 years	148.29	(142.63, 154.18)			
20–39 years	250.47	(237.2, 264.49)	< 0.001		
40–59 years	454.44	(439.55, 469.84)	< 0.001	< 0.001	
60+ years	1231.69	(1185.8, 1279.35)	< 0.001	< 0.001	< 0.001
Cycle 2009–2010					
12–19 years	124.09	(120.18, 128.12)			
20–39 years	210.84	(202.97, 219.02)	< 0.001		
40–59 years	392.85	(381.81, 404.22)	< 0.001	< 0.001	
60+ years	1053.84	(1021.68, 1087.01)	< 0.001	< 0.001	< 0.001
Cycle 2011–2012					
12–19 years	103.84	(100.15, 107.67)			
20–39 years	177.48	(172.86, 182.23)	< 0.001		

40–59 years	339.61	(329.48, 350.05)	< 0.001	< 0.001	
60+ years	901.68	(875.16, 928.99)	< 0.001	< 0.001	< 0.001
	Cycle 2013–2014				
12–19 years	86.89	(82.76, 91.23)			
20–39 years	149.4	(145.46, 153.45)	< 0.001		
40–59 years	293.58	(282.71, 304.87)	< 0.001	< 0.001	
60+ years	771.48	(744.87, 799.04)	< 0.001	< 0.001	< 0.001
	Cycle 2015–2016				
12–19 years	72.71	(68.14, 77.59)			
20–39 years	125.76	(120.98, 130.73)	< 0.001		
40–59 years	253.8	(241.83, 266.35)	< 0.001	< 0.001	
60+ years	660.09	(631.32, 690.16)	< 0.001	< 0.001	< 0.001
**Race* and Survey (*P* < 0.001)**			MA	NHB	NHW
	Cycle 2005–2006				
MA	1093.43	(973.41, 1228.25)			
NHB	188.55	(181.28, 196.11)	< 0.001		
NHW	340.38	(323.97, 357.63)	< 0.001	< 0.001	
OTHER	740.05	(671.65, 815.41)	< 0.001	< 0.001	< 0.001
	Cycle 2007–2008				
MA	890.9	(816.42, 972.17)			
NHB	163.94	(159.2, 168.82)	< 0.001		
NHW	289.2	(278.62, 300.18)	< 0.001	< 0.001	
OTHER	583.79	(544.93, 625.43)	< 0.001	< 0.001	< 0.001
	Cycle 2009–2010				
MA	725.88	(681.71, 772.91)			
NHB	142.54	(139.61, 145.53)	< 0.001		
NHW	245.71	(238.76, 252.87)	< 0.001	< 0.001	
OTHER	460.53	(438.32, 483.87)	< 0.001	< 0.001	< 0.001
	Cycle 2011–2012				
MA	591.43	(562.7, 621.62)			
NHB	123.93	(121.96, 125.94)	< 0.001		
NHW	208.77	(203.12, 214.57)	< 0.001	< 0.001	
OTHER	363.29	(345.59, 381.9)	< 0.001	< 0.001	< 0.001
	Cycle 2013–2014				
MA	481.88	(455.23, 510.08)			
NHB	107.75	(105.78, 109.76)	< 0.001		
NHW	177.38	(171.42, 183.54)	< 0.001	< 0.001	
OTHER	286.59	(267.21, 307.38)	< 0.001	< 0.001	< 0.001
	Cycle 2015–2016				
MA	392.62	(362.87, 424.81)			
NHB	93.69	(91.27, 96.17)	< 0.001		
NHW	150.7	(144, 157.73)	< 0.001	< 0.001	
OTHER	226.08	(204.91, 249.44)	< 0.001	< 0.001	< 0.001

*MA: Mexican American; NHB: Non–Hispanic black; NHW: Non–Hispanic white; OTHER: other than MA, NHB, and NHW

Table S5. LSGM (ng/g Lipid) and 95% CI by Demographic Groups for p, p'–DDT

Category	LSGM	95% CI	Pairwise comparison: *P*		
**Sex and Survey (*P* = 0.034)**					
	Cycle 2005–2006				

Male	6.07	(5.67, 6.5)			
Female	6.66	(6.32, 7)	0.016		
	Cycle 2007–2008				
Male	5.28	(5.01, 5.57)			
Female	5.65	(5.41, 5.89)	0.019		
	Cycle 2009–2010				
Male	4.59	(4.41, 4.78)			
Female	4.79	(4.62, 4.96)	0.04		
	Cycle 2011–2012				
Male	3.99	(3.85, 4.13)			
Female	4.06	(3.93, 4.2)	0.293		
	Cycle 2013–2014				
Male	3.47	(3.33, 3.61)			
Female	3.45	(3.33, 3.57)	0.757		
	Cycle 2015–2016				
Male	3.01	(2.86, 3.17)			
Female	2.92	(2.8, 3.05)	0.274		
**Age Group and Survey (*P* = 0.004)**			12–19 years	20–39 years	40–59 years
	Cycle 2005–2006				
12–19 years	2.99	(2.79, 3.2)			
20–39 years	5.83	(5.31, 6.4)	< 0.001		
40–59 years	7.39	(7.06, 7.75)	< 0.001	< 0.001	
60+ years	12.31	(11.64, 13.02)	< 0.001	< 0.001	< 0.001
	Cycle 2007–2008				
12–19 years	2.65	(2.52, 2.78)			
20–39 years	4.92	(4.58, 5.29)	< 0.001		
40–59 years	6.44	(6.2, 6.69)	< 0.001	< 0.001	
60+ years	10.41	(9.93, 10.9)	< 0.001	< 0.001	< 0.001
	Cycle 2009–2010				
12–19 years	2.35	(2.25, 2.45)			
20–39 years	4.15	(3.94, 4.37)	< 0.001		
40–59 years	5.61	(5.42, 5.81)	< 0.001	< 0.001	
60+ years	8.8	(8.45, 9.15)	< 0.001	< 0.001	< 0.001
	Cycle 2011–2012				
12–19 years	2.08	(1.98, 2.19)			
20–39 years	3.5	(3.36, 3.64)	< 0.001		
40–59 years	4.89	(4.72, 5.06)	< 0.001	< 0.001	
60+ years	7.44	(7.16, 7.72)	< 0.001	< 0.001	< 0.001
	Cycle 2013–2014				
12–19 years	1.84	(1.72, 1.98)			
20–39 years	2.95	(2.84, 3.07)	< 0.001		
40–59 years	4.26	(4.09, 4.42)	< 0.001	< 0.001	
60+ years	6.29	(6.03, 6.55)	< 0.001	< 0.001	< 0.001
	Cycle 2015–2016				
12–19 years	1.64	(1.48, 1.8)			
20–39 years	2.49	(2.36, 2.62)	< 0.001		
40–59 years	3.71	(3.54, 3.88)	< 0.001	< 0.001	
60+ years	5.31	(5.06, 5.58)	< 0.001	< 0.001	< 0.001
**Race* and Survey (*P* < 0.001)**			MA	NHB	NHW
	Cycle 2005–2006				
MA	14.88	(12.09, 18.31)			

NHB	2.93	(2.82, 3.04)	< 0.001		
NHW	5.16	(4.75, 5.6)	< 0.001	< 0.001	
OTHER	11.73	(10.28, 13.39)	0.122	< 0.001	< 0.001
Cycle 2007–2008					
MA	11.44	(9.81, 13.36)			
NHB	2.59	(2.52, 2.67)	< 0.001		
NHW	4.36	(4.1, 4.65)	< 0.001	< 0.001	
OTHER	9.25	(8.39, 10.2)	0.037	< 0.001	< 0.001
Cycle 2009–2010					
MA	8.8	(7.89, 9.82)			
NHB	2.3	(2.25, 2.35)	< 0.001		
NHW	3.69	(3.53, 3.86)	< 0.001	< 0.001	
OTHER	7.3	(6.79, 7.85)	0.01	< 0.001	< 0.001
Cycle 2011–2012					
MA	6.77	(6.21, 7.38)			
NHB	2.04	(2, 2.08)	< 0.001		
NHW	3.12	(3.01, 3.23)	< 0.001	< 0.001	
OTHER	5.76	(5.37, 6.17)	0.034	< 0.001	< 0.001
Cycle 2013–2014					
MA	5.21	(4.71, 5.76)			
NHB	1.81	(1.77, 1.86)	< 0.001		
NHW	2.64	(2.55, 2.74)	< 0.001	< 0.001	
OTHER	4.54	(4.15, 4.97)	0.306	< 0.001	< 0.001
Cycle 2015–2016					
MA	4.01	(3.47, 4.62)			
NHB	1.61	(1.55, 1.66)	< 0.001		
NHW	2.23	(2.13, 2.35)	< 0.001	< 0.001	
OTHER	3.58	(3.17, 4.05)	0.726	< 0.001	< 0.001

*MA: Mexican American; NHB: Non–Hispanic black; NHW: Non–Hispanic white; OTHER: other than MA, NHB, and NHW

Table S6. LSGM (ng/g Lipid) and 95% CI by Demographic Groups for trans–nonachlor

Category	LSGM	95% CI	Pairwise comparison: *P*
**Sex and Survey (*P* = 0.783)**			
Cycle 2005–2006			
Male	14.48	(13.79, 15.21)	
Female	12.87	(12.37, 13.39)	< 0.001
Cycle 2007–2008			
Male	13.2	(12.76, 13.67)	
Female	11.76	(11.42, 12.1)	< 0.001
Cycle 2009–2010			
Male	12.04	(11.77, 12.32)	
Female	10.74	(10.52, 10.96)	< 0.001
Cycle 2011–2012			
Male	10.98	(10.74, 11.21)	
Female	9.81	(9.64, 9.98)	< 0.001
Cycle 2013–2014			
Male	10.01	(9.7, 10.32)	
Female	8.96	(8.76, 9.17)	< 0.001
Cycle 2015–2016			

Male	9.12	(8.72, 9.55)			
Female	8.18	(7.92, 8.45)	< 0.001		
**Age Group and Survey (*P* < 0.001)**			12–19 years	20–39 years	40–59 years
Cycle 2005–2006					
12–19 years	4.79	(4.48, 5.13)			
20–39 years	8.44	(7.93, 8.98)	< 0.001		
40–59 years	19.7	(18.63, 20.82)	< 0.001	< 0.001	
60+ years	44.07	(41.86, 46.39)	< 0.001	< 0.001	< 0.001
Cycle 2007–2008					
12–19 years	4.28	(4.09, 4.49)			
20–39 years	7.54	(7.21, 7.88)	< 0.001		
40–59 years	18.24	(17.53, 18.99)	< 0.001	< 0.001	
60+ years	41.09	(39.55, 42.69)	< 0.001	< 0.001	< 0.001
Cycle 2009–2010					
12–19 years	3.83	(3.7, 3.97)			
20–39 years	6.73	(6.55, 6.93)	< 0.001		
40–59 years	16.9	(16.44, 17.37)	< 0.001	< 0.001	
60+ years	38.31	(37.22, 39.43)	< 0.001	< 0.001	< 0.001
Cycle 2011–2012					
12–19 years	3.42	(3.29, 3.57)			
20–39 years	6.02	(5.9, 6.13)	< 0.001		
40–59 years	15.65	(15.31, 16)	< 0.001	< 0.001	
60+ years	35.72	(34.75, 36.71)	< 0.001	< 0.001	< 0.001
Cycle 2013–2014					
12–19 years	3.06	(2.89, 3.25)			
20–39 years	5.37	(5.24, 5.51)	< 0.001		
40–59 years	14.5	(14.09, 14.92)	< 0.001	< 0.001	
60+ years	33.3	(32.17, 34.48)	< 0.001	< 0.001	< 0.001
Cycle 2015–2016					
12–19 years	2.74	(2.52, 2.97)			
20–39 years	4.8	(4.61, 5)	< 0.001		
40–59 years	13.43	(12.87, 14.01)	< 0.001	< 0.001	
60+ years	31.05	(29.62, 32.54)	< 0.001	< 0.001	< 0.001
**Race* and Survey (*P* = 0.00**5)			MA	NHB	NHW
Cycle 2005–2006					
MA	12.32	(11.55, 13.14)			
NHB	14.92	(14.01, 15.89)	< 0.001		
NHW	15.39	(14.74, 16.06)	< 0.001	0.849	
OTHER	13.4	(12.86, 13.96)	0.186	0.021	< 0.001
Cycle 2007–2008					
MA	11.27	(10.73, 11.85)			
NHB	13.71	(13.1, 14.36)	< 0.001		
NHW	13.97	(13.52, 14.43)	< 0.001	0.918	
OTHER	11.81	(11.43, 12.2)	0.492	< 0.001	< 0.001
Cycle 2009–2010					
MA	10.32	(9.93, 10.73)			
NHB	12.61	(12.22, 13)	< 0.001		
NHW	12.68	(12.36, 13)	< 0.001	0.992	
OTHER	10.41	(10.12, 10.7)	0.987	< 0.001	< 0.001
Cycle 2011–2012					
MA	9.45	(9.12, 9.78)			

NHB	11.59	(11.34, 11.85)	< 0.001		
NHW	11.51	(11.23, 11.79)	< 0.001	0.972	
OTHER	9.17	(8.91, 9.45)	0.571	< 0.001	< 0.001
Cycle 2013–2014					
MA	8.65	(8.31, 9)			
NHB	10.65	(10.37, 10.94)	< 0.001		
NHW	10.45	(10.13, 10.77)	< 0.001	0.689	
OTHER	8.09	(7.79, 8.39)	0.04	< 0.001	< 0.001
Cycle 2015–2016					
MA	7.92	(7.51, 8.34)			
NHB	9.79	(9.41, 10.19)	< 0.001		
NHW	9.49	(9.11, 9.88)	< 0.001	0.57	
OTHER	7.13	(6.8, 7.47)	0.006	< 0.001	< 0.001

*MA: Mexican American; NHB: Non–Hispanic Black; NHW: Non–Hispanic White; OTHER: other than MA, NHB, and NHW

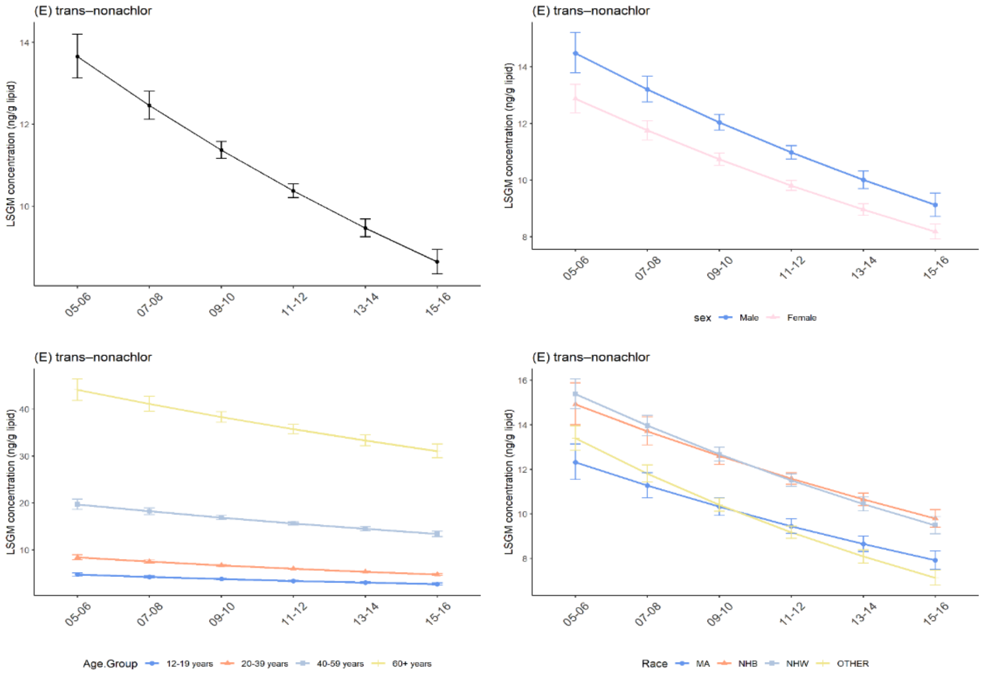

Figure S1. Temporal trends in serum trans–nonachlor concentration over survey cycles.
